# The Distribution Pattern and Leaching Toxicity of Heavy Metals in Glass Ceramics from MSWI Fly Ash and Andesite Tailings

**DOI:** 10.3390/toxics10120774

**Published:** 2022-12-10

**Authors:** Yongya Wang, Xinyi Huang, Wei Wang, Tao Wu

**Affiliations:** 1 School of Intelligent Manufacturing, Huzhou College, Huzhou 313000, China; 2 Department of Material Chemistry, Huzhou University, Huzhou 313000, China; 3 School of Environment, Tsinghua University, Beijing 100084, China

**Keywords:** glass ceramics, MSWI fly ash, leaching toxicity, heavy metal

## Abstract

The leaching of heavy metals (HMs) is the key factor affecting the resource utilization of municipal solid waste incineration (MSWI) fly ash. A novel fly ash and andesite-tailings-based (FAAT) glass ceramic is prepared with the full-component utilization of MSWI fly ash and andesite tailings. The effects of the content and distribution state of HMs on their leaching toxicity are studied by performing a sequential extraction procedure and leaching toxicity test. The results show that the MSWI fly ash content greatly impacts the HMs’ leaching toxicity in glass ceramics. Thus, the addition of MSWI fly ash must be maintained at below 20% so as to meet the class III groundwater standard. Furthermore, the different distribution states of Zn and Cr also affect their leaching toxicity. Zn suits the requirements for leaching toxicity only in a 2080c sample, while Cr fulfills the class III groundwater standard for all the glass ceramics. Since this finding is mismatched with the calculated potential ecological risk index of glass ceramics, the latter can only be used as a reference. Therefore, the results of the present study are of great significance in the vitrification application of MSWI fly ash.

## 1. Introduction

The recycling and disposal of industrial solid waste tailings and municipal solid waste incineration (MSWI) fly ash has become a common concern across the world [1,2]. Less damaging ways to process municipal solid waste and a reduction in the hazardous impact of waste disposal on the environment are urgently needed [3]. In this respect, melting is one of the most popular methods used to stabilize heavy metals in MSWI fly ash, while dioxin and other organic pollutants are decomposed and destructed completely [4,5]. In particular, treatment gasifies heavy metals (HMs) in MSWI fly ash or transforms them to glassy slag [6,7]. As a result, the apparent volume of MSWI fly ash is greatly reduced, the HMs are stabilized to achieve their harmless effect, and the glass slag can be reused [8]. Therefore, melting would be the most promising method of MSWI fly ash processing [9,10,11].

MSWI fly ash is rich in CaO and contains some amounts of Al_2_O_3_ and SiO_2_. Glass slag after melt treatment can transform into glass ceramics and other materials through appropriate raw material selection and recrystallization, which is due to its loose structure and insufficient long-term stability [12,13]. In turn, andesite tailings are mainly composed of SiO_2_. The combination of MSWI fly ash and andesite tailings would help to meet the requirements for glass ceramics composition [14]. Moreover, the addition of silicon rich tailings seems to be a reliable route to decrease the melting point of MSWI fly ash and to reduce its energy consumption [15,16,17]. Therefore, the preparation of glass ceramics using MSWI fly ash and andesite tailings is a feasible path for their efficient resource utilization.

Compared with traditional glass products, the glass matrix consisting of numerous grains plays a dual role in the stability of HMs in glass ceramics, effectively alleviating the problem of re-leaching of HMs caused by changes in environmental conditions [18]. However, when used as building materials, glass ceramics must meet the stringent requirements for leaching toxicity of HMs. Therefore, the leaching toxicity of HMs in fly ash-based glass ceramics has been studied for a long time [19]. For example, a thorough analysis of glass from MSWI fly ash revealed a large amount of phosphate and silicate that were assumed to be responsible for the stability of the HMs [20]. Moreover, the distribution characteristics of HMs were found to depend on the evaporative properties [21]. In turn, the existing forms of HMs impact stabilization performance and leaching toxicity [22]. The HM ions can replace other ions by forming substitutional solid solutions and occupying the corresponding sites in the crystal structure of glass ceramics [5,23]. The leaching behavior of HMs suggests that their immobilization in the activated mixtures might be due to both physical and chemical mechanisms [24]. So, the dominant leaching mechanism was surface wash off in the initial stages followed by diffusion for HMs [25].

The present work aims at the efficient treatment and disposal of MSWI fly ash and andesite tailings. For this, fly ash and andesite tailings-based (FAAT) glass ceramics were prepared using both solid wastes as the raw materials. The leaching toxicity of HMs in FAAT glass and glass ceramics was investigated. The solidification mechanism of HMs in FAAT glass ceramics was discussed based on the analysis of the content, distribution and morphology of HMs. The results of this study provide a theoretical basis for the effective reuse of MSWI fly ash and andesite tailings.

## 2. Materials and Methods

### 2.1. Preparation of the Glass–Ceramic Samples

**Figure 1:**
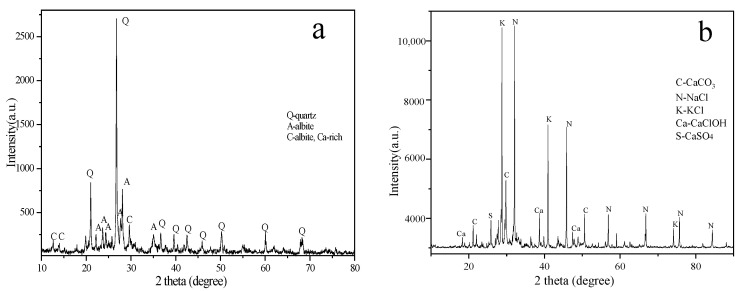
XRD patterns obtained from andesite tailing (**a**) and MSWI fly ash (**b**).

Andesite tailings with quartz and albite main crystal phases were taken from a mine in Huzhou City, China. The MSWI fly ash was collected from a municipal solid waste incineration plant in Tianjin City, China. The major chemical composition of each material determined by XRF is shown in Table 1, and the corresponding XRD patterns are shown in Figure 1.

The raw materials were prepared with respect to the specific ratios established for each formula, as shown in Table 2. The raw materials were labelled as xxxxr, the glass samples were referred to as xxxxg, and the glass–ceramic specimens were denoted as xxxxc. The andesite tailings and MSWI fly ash powders were ground using a 200-mesh sieve and mixed in a corundum crucible. Then, they were molten at 1450 °C for 2 h in a high temperature muffle furnace; liquid glass was quenched with water into glass slags, which were dried at 120 °C for 6 h and ground through a 200-mesh sieve. The obtained basic glass powder was evenly stirred with a small amount of distilled water and placed into a circular mold. A pressure of 20 MPa was applied for 5 min to create a green body that was subsequently dried at 120 °C for 8 h. Afterwards, the green body was placed in a muffle furnace and heated to 690 °C for 30 min at a heating rate of 5 °C/min. The heating temperature was then increased to 970 °C at a heating rate of 2 °C/min and maintained for 2.5 h. The products were finally cooled to room temperature to obtain glass ceramics.

### 2.2. Characterization

The microstructure of the samples was observed via scanning electron microscopy (SEM) using a MERLIN VP Compact system (Zeiss, Oberkochen, Germany). Prior to the SEM analysis, the samples were coated with an approximately 10 nm thick Pt layer. Focused ion beam secondary ion mass spectrometry (FIB-SIMS) was applied by means of a S9000X (TESCAN, Shanghai, China) installation for high resolution element surface analyses of specimens.

The phase composition analysis was performed via X-ray diffraction (XRD). XRD diffractograms were collected within a 2θ range of 10–80° using a D/max-2550 X-ray diffractometer (Rigaku, Tokyo, Japan) operating at 36 kV and 20 mA. Prior to the experiments, the samples were ground and passed through a 300-mesh sieve. The relative masses of each phase were calculated using the K value method with Jade 6 software(MDI, California, USA). The melting points of the specimens were measured with a HKHR-4000 melting point meter (Hengke, Hebi, China). The calculation method of the alkalinity and weight-loss rate is as follows:

(1) $$Alkalinity=\frac{w_{CaO}}{W_{SiO2}}$$

(2) $$\;Weight\;loss\;rate=\frac{W_{raw}-W_{gc}}{W_{raw}}\times 100\%$$

$w_{CaO}$ and $W_{SiO2}$ are the content of CaO and SiO_2_ in glass ceramics determined by XRF; $W_{raw}$ and $W_{gc}$ are the weight of raw materials and corresponding glass–ceramic samples.

The chemical composition of the samples was analyzed via X-ray fluorescence (XRF) spectrometry using ARL PERFORM X equipment (Thermo, Waltham, USA), while the HM contents (Ni, Zn, Mn, Cr, Pb, Cu, Cd, As, Co, Ti and Hg) were determined via inductively coupled plasma mass spectrometry (ICP-MS) on a Thermo, iCAP RQ system (Thermo) according to HJ 776-2015 of Water Quality-Determination of 32 elements-Inductively coupled plasma optical emission spectrometry. The potential ecological risk index (RI) was used to evaluate the pollution level of HMs according to the Hakanson formula, as follows [26,27]:

(3) $$RI=\sum^{\;}E_{r}^{i}$$

where

(4) $$E_{r}^{i}=T_{r}^{i}\times C_{f}$$

and

(5) Cf=w(i)Cn

*C_f_* is the pollution factor of a single HM element; w(i) is the mass fraction of HMs i in the glass or glass ceramic samples; $C_{n}$ is the background value of HMs; $T_{r}^{i}$ is the toxicity factor of the i-th HM element; and $E_{r}^{i}$ is the potential ecological risk index of the i-th HM. RI is the sum of potential ecological risk indexes of several HMs.

The HM distribution patterns in samples were determined using a modified sequential extraction procedure developed by Tessier et al. [28,29]. For this, 5 g of the glass and glass ceramic powder samples were placed into a 50 mL polyethylene vessel and an extraction solution was added. The blend was stirred using a rotation machine and heated. The extraction conditions are summarized in Table 3. After extracting each fraction, the extraction solution was filtered through the 0.45 μm membranes. The metal concentrations of each fraction were analyzed using ICP-MS.

The leaching testing of samples was carried out following Method 1311 of the Toxicity Characteristic Leaching Procedure (TCLP) proposed by US EPA [30]. The leaching fluid was an acetic acid solution with pH of 4.93 (extraction fluid #1, Method 1311, US EPA). Prior to testing, 80 g of the powder sample and 1600 mL of the extraction fluid were mixed in a leaching vial and rotated adequately for 18 h with a rotary tumbler at a rotation speed of 30 ± 2 rpm. The leaching concentrations of HMs were analyzed using an ICP-MS spectrometer (Thermo, iCAP RQ) after being filtered through a 0.45 μm filter membrane. The leaching toxicity of the samples was then compared with the data provided by the class III groundwater standard (GB/T 14848-2017, standard for groundwater quality). The standard specifies the quality, classification, indicators and limits of groundwater. The class III groundwater is mainly applicable to centralized domestic drinking water sources and industrial and agricultural water. The concentration of metals according to the class III groundwater standard are listed in Table 4. For this, the leaching ratios $L_{k}^{i}$ of the HMs were defined as follow:

(6) $$L_{k}^{i}=\frac{m_{k}^{\prime }}{m_{k}^{0}}\times 100\%$$

where $m_{k}^{\prime }$ and $m_{k}^{0}$ are the masses of the i-th HM element in leachate and the solid sample, respectively.

## 3. Results

### 3.1. Phase Analysis and Physical Properties Characterization

**Figure 2:**
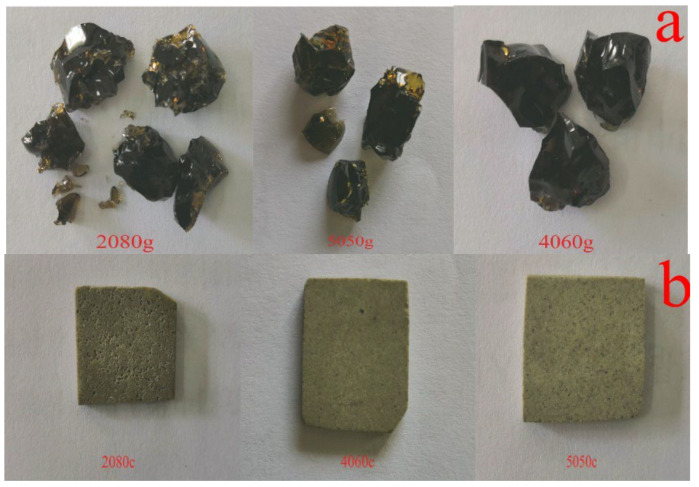
Photographs of glass (**a**) and glass ceramic (**b**) samples.

**Figure 3:**
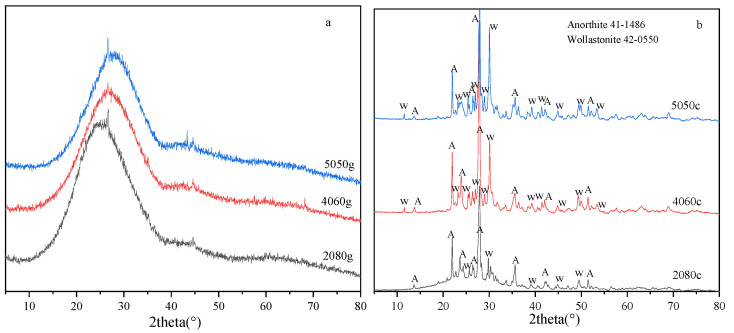
XRD patterns obtained from the glass (**a**) and glass ceramic (**b**) samples.

As seen in the photographs in Figure 2a, all the samples formed translucent glasses after melting treatment. The XRD halo at about 27°, attributed to glass, is also observed in the XRD patterns in Figure 3a. According to the optical images in Figure 2b, translucent glasses become opaque glass ceramics after secondary heat treatment, and the corresponding XRD patterns are shown in Figure 3b.

The major crystalline phases of the glass ceramic samples were anorthite (JCPDS No. 41-1486) and wollastonite (JCPDS No. 42-0550), and there were also some glassy states in the samples. The relative masses of each phase calculated from the XRD patterns are listed in Table 5. It is evident that the amount of wollastonite gradually increased while the content of anorthite decreased with an increasing degree of alkalinity. This is mainly because the activation energy of wollastonite (261.99 kJ/mol) is lower than that of anorthite (308 kJ/mol) [31]. Therefore, a large quantity of calcium introduced from MSWI fly ash was combined with feldspar from the tailings to form wollastonite and alumina, allowing alumina to achieve a glassy state [32].

The alkalinity, melting points and weight-loss rates after heat treatment of the samples are also listed in Table 5. The alkalinity of the sample increased with the increase in the MSWI fly ash content in the sample while the melting point of the sample gradually decreased. This was mainly due to the decrease in the quartz concentration caused by the introduction of andesite tailings that also led the melting point to reduce to a certain extent. At the same time, most of the chlorides and metals with low melting points volatilized during melting, leading to the formation of secondary fly ash and to the increase in the weight-loss rate of the sample. In addition, the melting point of the sample gradually stabilized with the increase in alkalinity to 0.5; however, the weight-loss rate continued to rise. Therefore, the content of MSWI fly ash should be controlled so as to reduce the weight-loss rate and the production of secondary fly ash and maintain a low melting point.

### 3.2. Major Chemical Compositions and HMs Concentrations of the Samples

The major chemical compositions of raw materials and samples determined by XRF are listed in Table 6. According to the data, the S and Cl elements almost completely volatilized and the contents of K and Na also decreased dramatically during heat treatment. In turn, the amounts of other elements changed slightly or even increased. Finally, the mass of base glass and its main elements remained almost unchanged before and after secondary heat treatment.

The HMs concentrations in raw materials and samples evaluated via ICP-MS are given in Table 7. Based on these results, the contents of different HMs changed greatly with varying raw material compositions. Moreover, metal Co was not detected in the samples and the amount of Hg was also very low; therefore, their leaching toxicities can be ignored and will not be discussed below. All the materials under consideration were found to be rich in Zn, Mn, Cr, Pb and Cu, among which Zn is a low-melting-point metal and Cr is a high-melting-point metal. Therefore, the content of Zn noticeably decreased during the vitrification process, whereas the absolute quantity of Cr basically remained unchanged. Subsequently, these two metals were chosen as representative elements for further discussion. Similar to the contents of major chemical elements, those of other HMs decreased significantly during vitrification (except Cr), but they hardly varied upon secondary heat treatment. Thus, it can be assumed that the content of HMs in basic glass and glass ceramics was basically the same.

### 3.3. Morphology and Element Distribution of the Samples

**Figure 4:**
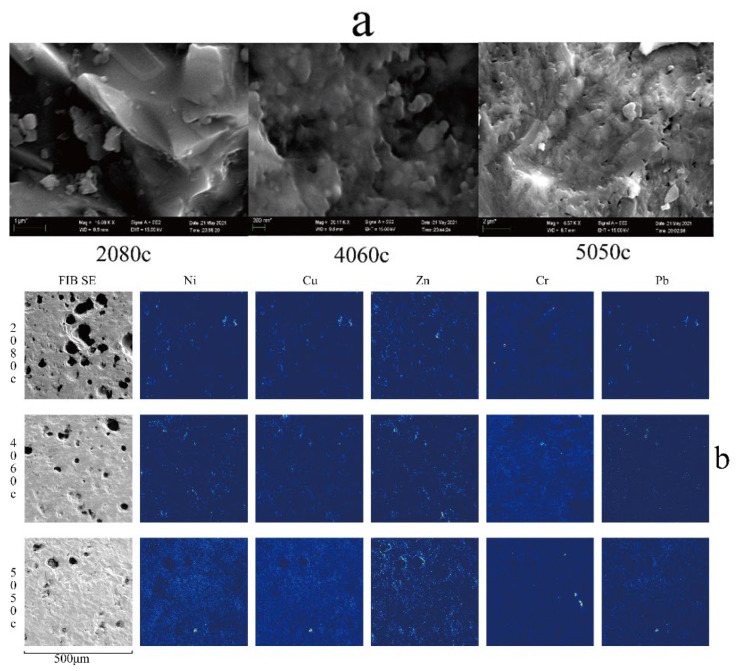
(**a**) SEM micrographs and (**b**) FIB-SIMS images of the glass ceramic samples.

Figure 4a displays the SEM micrographs revealing the microstructural features of the glass ceramic samples. In all cases, flakes or granules with an irregular shape were observed. In particular, the 2080c specimen exhibited a dense structure with short and thick grains. In the 4060c and 5050c samples, granules were connected together through a glass state. However, the difference is that grains composing sample 4060c were spherical, while those in the 5050c specimen were flaky.

Figure 4b shows the FIB-SIMS images of glass ceramics. The overlaps between the distribution images of Ni and Cu suggested that both elements were likely to exist in glass ceramics in the form of isomorphous replacements. The contents measured by ICP-MS were found to be basically the same, which agrees with the above conclusion to a certain extent. In turn, the Zn and Cr elements with high contents had completely different distribution states. While Zn elements were uniformly distributed, the Cr elements were relatively discrete and scattered across the ceramic structure. This means that the occurrence states of Zn and Cr in glass ceramics were completely different, which might have exerted a certain impact on the leaching toxicity of these HMs.

### 3.4. The HMs Distribution Patterns in the Samples

**Figure 5:**
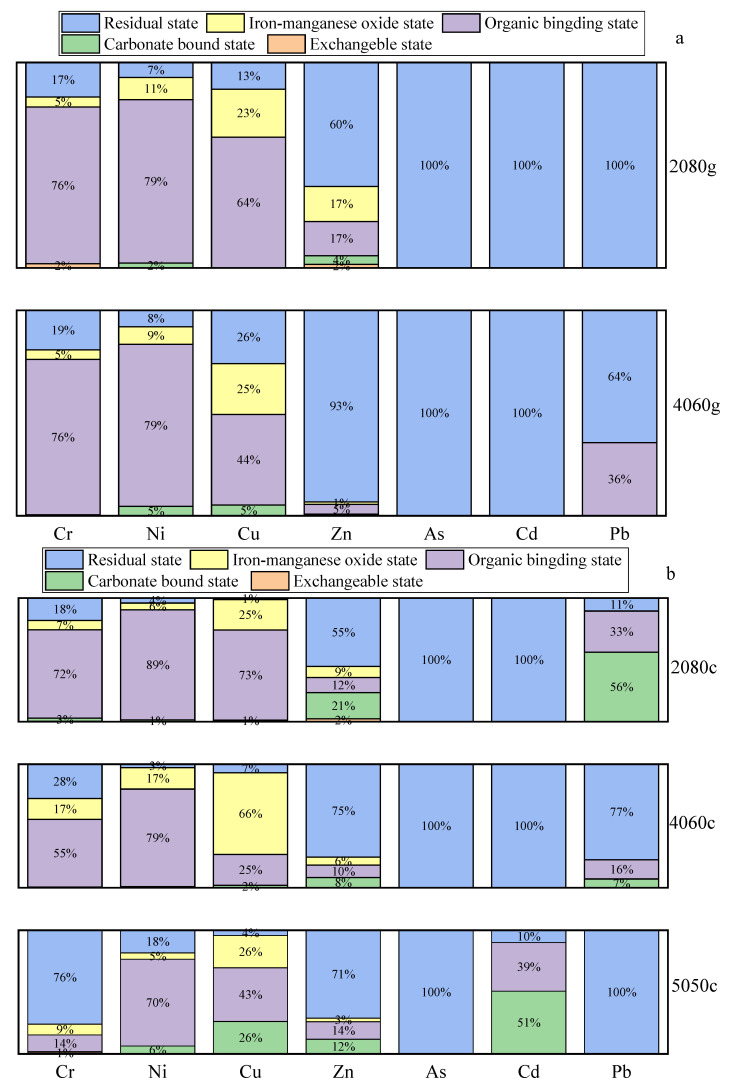
HM distribution patterns in (**a**) glass and (**b**) glass ceramic samples.

Figure 5 displays the HMs distribution patterns in glass (Figure 5a) and glass ceramic (Figure 5b) samples. According to the results, almost no HMs existed in exchangeable state, while As, Cd and Pb were basically in a residual state. With the increase in the MSWI fly ash content, the amount of Cr and Zn in a residual state increased gradually, whereas that of Cr elements in an organic binding state and Zn in a carbonate-bound state decreased. Compared with glass samples, the quantities of Ni, Cu and Zn in residual states in specimens 2080c and 4060c were reduced, while the content of residual Cr increased and that of the organic binding Cr decreased. The carbonate-bound state of most HMs increased in the glass ceramic sample relative to that in the glass sample.

### 3.5. The Potential Ecological Hazard Indices of HMs

**Figure 6:**
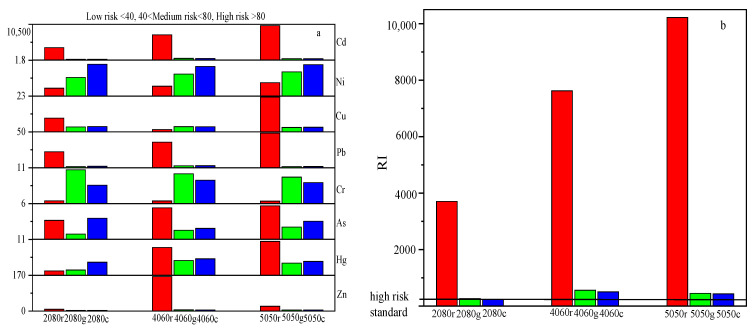
(**a**) Single HM potential ecological risk level and (**b**) multi metal potential ecological risk index (RI).

Figure 6a depicts the potential ecological hazard indices of different metals calculated according to Hakanson Formulas (3) and (4). The background value in the calculation was taken from the mass fraction of HMs (mg/kg) according to the GB15618-1995 first-class standard for soils.

According to these data, the potential ecological hazard indices of Pb, As, Hg, Cu, Zn and Cd decreased after vitrification treatment, and all the metals except Cd exhibited low ecological risk levels. Moreover, the potential ecological hazard indices of single HMs in both the glass and glass ceramic samples were almost the same. In turn, the RI diagrams of the samples are plotted in Figure 6b. The lowest RI value of the raw material was 3700, while the highest one for the glass and glass–ceramic samples was 560. Therefore, the potential ecological hazard index of various metals in glass ceramics was at a relatively reduced level. Although the RI values of the glass and glass–ceramic samples still reflected their high-risk level, the latter value, however, was much lower than the potential ecological risk of the raw material.

### 3.6. The Leaching Toxicities and Leaching Rates of HMs

**Figure 7:**
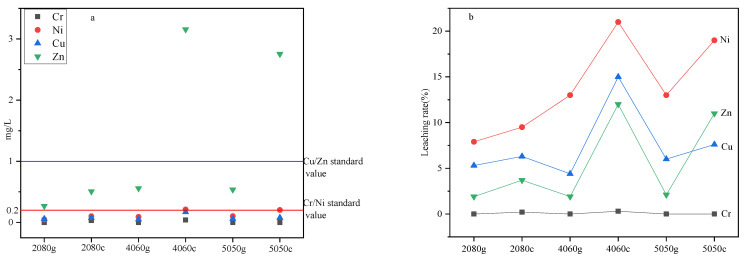
(**a**) Leaching toxicity and (**b**) leaching rates of HMs in glass and glass ceramic samples.

Figure 7a displays the leaching toxicities of the glass and glass ceramic samples in which As, Cd, Pb and Hg are not detected. According to the results, the HM leaching toxicity of all glass samples was found to be within the class III groundwater standard. As for glass ceramics, the HM leaching toxicity of the 2080c specimen fulfilled the mentioned standard as well. In turn, the level of Zn in samples 4060c and 5050c exceeded the recommended amount, while other HMs remained at the acceptable level. Therefore, the leaching toxicity of HMs in glass ceramic samples could be improved to various degrees compared with glass samples, and the corresponding leaching rates of HMs (Figure 7b) were found to be basically the same as the leaching toxicity. In particular, the leaching rate of Cr in glass ceramics was substantially lower than that of Zn. In turn, the leaching rates of HMs in glass were inferior to those in glass ceramics. This seems to be related to the change of the occurrence states of HMs in glass ceramics compared with glass samples.

## 4. Discussion

The calculated potential ecological hazard indices showed that Cr and Zn were at the low ecological risk level. However, according to the leaching toxicity results, the leaching toxicity of Zn in glass ceramics 4060c and 5050c exceeded that recommended by class III groundwater standards once the MSWI fly ash content increased. Meanwhile, other HMs were able to meet the mentioned standards. At the same time, the leaching toxicity of all glass samples was found to be within the standard level, and those of Cr and Zn in the 2080g, 4060g and 5050g specimens varied to a small extent. This was explained by different occurrence states of Cr and Zn in glass and glass ceramics.

The residual state content of Cr in the 2080c, 4060c and 5050c samples gradually increased with the increase in the MSWI fly ash content, and the amount of Cr elements in the organic binding state correspondingly decreased. However, the leaching toxicity of Cr in the glass ceramics sample was not changed greatly, which indicated that Cr was in a stable state in glass ceramics. Furthermore, the presence of Cr could promote phase separation and induce crystallization. Moreover, the strong electric field of Cr was conducive to the transformation from a disordered to ordered structure and to the reduction in the potential energy barrier of crystal formation [33]. Moreover, the silica network was destroyed by Cr during the nucleation of the precursor glass, while a large number of core points were formed and matured in the subsequent crystallization heat treatment [34]. This led most of the Cr to form the Cr_2_O_3_ crystal structure.

The internal energy of the Cr_2_O_3_ crystal was low and its structure was stable. In addition, the chemical bonds between the elements led Cr to solidify well [35]. Subsequently, the crystalline phase was wrapped by a layer of glass with strong acid resistance, which effectively prevented contact between the Cr_2_O_3_ phase and the acid solution [36]. According to the results on leaching rates, more than 99.7% Cr could stably exist in the glass ceramic sample, and its leaching rate was less than 0.3%, which agreed with the above findings.

Although the content of residual Zn elements increased to a certain extent, the amount of Zn in an organic binding state was almost unchanged, whereas that in the iron-manganese oxide state and carbonate-bound state were reduced. The leaching toxicity of Zn in the glass ceramic sample also greatly increased. This finding could be explained as follows. The effective ion radius of Zn is small, while the binding force with oxygen ions is strong; therefore, it is easy to break the glass network [37]. Zn was mainly incorporated into wollastonite structures by substituting ferrous ions because of their similar partial positive charge and anion radii [38]. As a result, most Zn elements existed in the crystal phase in the form of isomorphous replacements, while a small amount was found in a glass matrix in the form of a solid solution [39,40]. Since wollastonite is prone to surface crystallization, most Zn elements are distributed on the surface of glass ceramics [35]. Therefore, the leaching toxicity of Zn is much greater than that of Cr. In addition, the content of Zn elements in samples 4060c and 5050c was nearly twice that of the 2080c specimen, which might have also increased the leaching toxicity.

In conclusion, the calculated potential ecological hazard indices showed that Cr and Zn in glass and glass ceramics samples were at the low ecological risk level. However, the amounts and occurrence states of HMs were found to affect the phase composition and distribution as well as the metal leaching behavior of the glass ceramic products. Zn mainly existed in the surface crystallized wollastonite crystal in the form of isomorphic substitution, resulting in the increase in leaching toxicity in some glass ceramics samples to over the level recommended by the class III groundwater standards. At the same time, the leaching toxicity of Cr in all the glass ceramics met the standards.

## 5. Conclusions

A novel FAAT glass ceramic was prepared with full component utilization of MSWI fly ash and andesite tailings. Although Cr and Zn in glass ceramics samples were found to be generally at the low ecological risk level, the leaching toxicity of Zn in the 4060c and 5050c samples exceeded the class III groundwater standards, while that of Cr in all the glass ceramics was at the acceptable level. This means that the distribution states of HMs could affect their leaching toxicity and the calculated potential ecological hazard indices would only serve as reference. The findings of this work provide a method for the efficient recycling of MSWI fly ash and andesite tailings to greatly reduce their hazardous impact on the environment.

## Figures and Tables

**Table 1:** Major chemical composition of raw materials (wt%).

Raw Materials	Na_2_O	MgO	Al_2_O_3_	SiO_2_	P_2_O_5_	SO_3_	Cl	K_2_O	CaO	Fe_2_O_3_	ZnO	TiO_2_	PbO
Fly ash	9.41	1.08	0.765	3.08	0.423	6.17	20.84	6.77	47.82	1.24	0.91	0.41	0.136
Andesite tailings	0.62	0.38	14.80	55.30	0.04	0.17	-	9.64	8.50	9.29	0.08	-	0.47

**Table 2:** Components of the glass–ceramic samples (wt%).

Sample	MSWI Fly Ash	Andesite Tailings
2080r	20	80
4060r	40	60
5050r	50	50

**Table 3:** Sequential extraction procedures.

Step	State	Extraction Solution	Mixing Time (h)	Temperature (°C)
F1	Exchangeable	100 mL 1 M NaOAc, pH = 8.2	1	27
F2	Carbonate bound	100 mL 1 M NaOAc, pH = 5	5	27
F3	Fe-Mn oxides	100 mL 0.04 M NH_2_OH·HCl in 25% HOAc	5	96 ± 3
F4	Organic binding	15 mL 0.02 M HNO_3_ + 40 mL 30% H_2_O_2_ 25 mL 3.2 M NH_4_OAc in 20% HNO_3_	5 0.5	85 ± 2 27
F5	Residual	10 mL HNO_3_, 5 mL HClO_4_, 10 mL HF, digested		

**Table 4:** The concentration of metals according to the class III groundwater standard.

HMs	Cu	Zn	Cr	Ni	As	Pb	Cd
Concentration (mg/L)	1	1	0.2	0.2	0.01	0.01	0.005

**Table 5:** Property parameters of the samples.

Sample	Anorthite (wt%)	Wollastonite (wt%)	Alkalinity	Melting Point (°C)	Weight Loss Rate (%)
2080	90.1	9.9	0.2	1376	23.54
4060	88.8	11.2	0.5	1181	27.95
5050	66.1	33.9	0.7	1165	33.08

**Table 6:** Major chemical composition of raw materials and samples determined by XRF (wt%).

Sample	Na_2_O	MgO	Al_2_O_3_	SiO_2_	SO_3_	Cl	K_2_O	CaO	Fe_2_O_3_
2080r	2.54	0.725	15.32	53	1.3	4.07	5.55	12.07	4.08
2080g	2.25	0.894	16.05	55.97	0.179	0.801	4.45	13.27	4.74
2080c	2.18	0.812	16.19	55.68	0.23	0.763	4.42	13.43	4.87
4060r	3.51	0.764	12.14	42.98	2.46	7.56	5.82	19.4	3.59
4060g	2.05	1.24	15.64	47.41	0.135	0.872	2.24	24.29	4.34
4060c	1.92	1.14	15.78	47.69	0.242	0.969	2.28	23.8	4.44
5050r	4.51	0.887	8.65	30.95	3.59	11.85	6.14	28.4	2.88
5050g	1.56	1.4	16.37	42.5	0.101	1.07	1.27	29.86	3.96
5050c	1.53	1.32	16.36	43.15	0.214	1.29	1.29	29.19	4.07

**Table 7:** The HMs contents of raw materials and samples determined by ICP-MS (ppm).

Sample	Zn	Ni	Cu	Pb	Cr	Cd	As	Co	Ti	Mn	Hg
2080r	859	5.29	61.5	156	37.3	24.4	4.79	N.D.	3.53	953	0.005
2080g	282	12.4	22	11.6	466	1.59	1.32	N.D.	3.7	956	0.006
2080c	275	21.3	24	14	254	1.49	5.3	N.D.	3.68	964	0.015
4060r	16,400	6.57	10.6	250	36	49.4	7.93	N.D.	2.94	768	0.032
4060g	575	14.7	23.3	18.8	411	3.55	2.28	N.D.	4.06	886	0.017
4060c	529	19.8	22.8	19.8	323	3.17	2.77	N.D.	2.18	857	0.019
5050r	2260	8.95	156	339	33	67.4	N.D.	N.D.	4.76	633	0.039
5050g	500	16.2	20	11.9	366	2.85	3.09	N.D.	3.33	780	0.014
5050c	502	21	20.5	12.8	289	2.73	4.56	N.D.	2.74	777	0.016

## Data Availability

Not applicable.
